# Substantial variation in the timing of pollen production reduces reproductive synchrony between distant populations of *Pinus sylvestris* L. in Scotland

**DOI:** 10.1002/ece3.3154

**Published:** 2017-06-15

**Authors:** Richard Whittet, Stephen Cavers, Joan Cottrell, Cristina Rosique‐Esplugas, Richard Ennos

**Affiliations:** ^1^ Institute of Evolutionary Biology University of Edinburgh Edinburgh UK; ^2^ NERC Centre for Ecology and Hydrology Penicuik UK; ^3^ Forest Research Northern Research Station Roslin UK

**Keywords:** assortative mating, countergradient variation, cumulative link model, flowering phenology, functional connectivity, gene flow, *Pinus sylvestris*, pollen, reproductive synchrony, Scotland

## Abstract

The ability of a population to genetically adapt to a changing environment is contingent not only on the level of existing genetic variation within that population, but also on the gene flow received from differently adapted populations. Effective pollen‐mediated gene flow among plant populations requires synchrony of flowering. Therefore differences in timing of flowering among genetically divergent populations may reduce their ability to adapt to environmental change. To determine whether gene flow among differently adapted populations of native Scots pine (*Pinus sylvestris*) in Scotland was restricted by differences in their flowering phenology, we measured timing of pollen release among populations spanning a steep environmental gradient over three consecutive seasons (2014–2016). Results showed that, over a distance of 137 km, there were as many as 15.8 days’ difference among populations for the predicted timing of peak pollen shedding, with the earliest development in the warmer west of the country. There was much variation between years, with the earliest development and least synchrony in the warmest year (2014) and latest development and greatest synchrony in the coolest year (2015). Timing was negatively correlated with results from a common‐garden experiment, indicative of a pattern of countergradient variation. We conclude that the observed differences in reproductive synchrony were sufficient to limit gene flow via pollen between populations of *P. sylvestris* at opposite ends of the environmental gradient across Scotland. We also hypothesize that continually warming, or asymmetrically warming spring temperatures will decrease reproductive synchrony among pine populations.

## 1 INTRODUCTION

A characteristic of many boreal and northern temperate tree species is the capacity for long‐distance pollen dispersal by wind, and high levels of gene flow between populations are thought to be widespread (Kremer et al., 2012; Savolainen, Pyhäjärvi, & Knürr, 2007). Gene flow among small remnant populations of trees is essential for maintenance of the naturally high levels of genetic variation within populations and provides the raw material upon which natural selection can act to enable populations to continually adapt to environmental changes (Davis & Shaw, 2001).

*Pinus sylvestris* L. is one such species capable of extensive pollen dispersal over long distances (Robledo‐Arnuncio & Gil, 2005; Varis, Pakkanen, Galofré, & Pulkkinen, 2009). In Scotland, *P. sylvestris* persists in 84 fragmented semi‐natural populations, also known as the “Caledonian pinewoods,” thought to represent only 1% of its former maximum distribution (McVean & Ratcliffe, 1962). Despite this severe fragmentation, levels of neutral genetic variation remain similar to those observed in more continuous parts of the species range in Eurasia, with the majority of the genetic variation held within rather than between populations (Forrest, 1980; Forrest, 1982; Kinloch, Westfall, & Forrest, 1986; Provan et al., 1998; Wachowiak, Iason, & Cavers, 2013; Wachowiak, Salmela, Ennos, Iason, & Cavers, 2011). One possible explanation for the low level of genetic differentiation among populations is that they are or have recently been connected by high levels of gene flow.

Despite the lack of neutral genetic population structure, there is evidence from common‐garden experiments that these populations are genetically differentiated for a range of adaptive traits (Donnelly, Cavers, Cottrell, & Ennos, 2016; Perry, Brown, Cavers, Cottrell, & Ennos, 2016; Perry, Wachowiak, et al., 2016; Salmela, Cavers, Cottrell, Iason, & Ennos, 2011, 2013), indicating that spatially variable selection is sufficiently strong to counteract the homogenizing effect of gene flow. An adaptive trait which has been shown to vary among populations is spring vegetative phenology (timing of bud burst). Under common‐garden conditions in a glasshouse, saplings from populations from colder environments initiated annual growth earlier than those from warmer environments (Salmela et al., 2013). Differentiation for spring phenology is common in trees, typically showing moderate to high *Q*_*ST*_ (a measure of differentiation in trait means among populations) in response to clines in temperature (Alberto et al., 2013; Gömöry et al., 2015; Le Corre & Kremer, 2012). The phasing of initiation and cessation of annual growth evolves as a mechanism by which to maximize annual growth whilst minimizing the risk of frost damage in spring and autumn (Aitken, Yeaman, Holliday, Wang, & Curtis‐McLane, 2008; Howe et al., 2003; Lenz, Hoch, Körner, & Vitasse, 2016; Vander Mijnsbrugge, Onkelinx, & De Cuyper, 2015). Due to its high adaptive genetic differentiation, selective and silvicultural importance, and relative ease of assessment from a young age, spring vegetative phenology is frequently assessed in provenance tests (Aitken & Bemmels, 2016; Alberto et al., 2013).

Reproductive phenology (i.e., timing of flowering in angiosperms, or timing of strobilus development in gymnosperms) is more difficult to investigate in common‐garden experiments because many tree species have delayed maturity (Petit & Hampe, 2006). One consequence of delayed maturity is that reproductive phenology does not come under selection for several years after establishment (Vander Mijnsbrugge et al., 2015). There can theoretically be many reproductive events in the lifetime of an individual tree. The penalties for poorly timed reproductive output are lower than penalties for poorly timed growth, which can include mortality due to exposure to frost. Therefore, selection on reproductive phenology is likely to be weaker than on timing of bud burst giving rise to higher levels of phenotypic plasticity than that observed for timing of bud burst which is under selection from a very young age (Koski & Sievänen, 1985; Vander Mijnsbrugge et al., 2015). However, reproductive phenology is almost certainly serially autocorrelated with the timing of bud burst (Soularue & Kremer, 2012, 2014) and is highly relevant for population and landscape genetic studies which aim to understand patterns of gene flow, local adaptation, and genetic structure (Kremer et al., 2012; Manel, Schwartz, Luikart, & Taberlet, 2003; Ramstad, Woody, Sage, & Allendorf, 2004; Thomasset et al., 2014).

In *P. sylvestris*, there is good evidence for variation in reproductive phenology among populations. The majority of this evidence has been generated from research in seed orchards in Fennoscandia (especially Finland) which is motivated by a need to understand pollen contamination of trees in selection and improvement programs (Jonsson, Ekberg, & Eriksson, 1976; Chung, 1981; Parantainen & Pulkkinen, 2003), and from forest stands in situ (Luomajoki, 1993; Parantainen & Pulkkinen, 2002; Pulkkinen & Rantio‐Lehtimäki, 1995; Varis et al., 2009). A common finding from these studies is that pollen tends to be produced in the warmer south of Finland earlier than in the colder north but that there can be considerable interannual variation in timing.

Although there are data on the timing of spring vegetative phenology from a glasshouse experiment (Salmela et al., 2013), no information on the timing of pollen production in Scottish pinewoods in situ yet exists. Therefore, the aims of this study were to investigate whether there were differences in the timing of pollen production among native populations of *P. sylvestris* in Scotland in situ, and whether these differences were maintained across three consecutive years (2014; 2015; 2016). We consider whether the degree of synchrony in reproductive phenology observed between populations in different environments could limit long‐distance gene flow and compare our observations with the extent of genetic connectivity suggested by previous marker‐based studies (Forrest, 1980; Kinloch et al., 1986; Provan et al., 1998; Wachowiak et al., 2011, 2013).

## 2 MATERIALS AND METHODS

### 2.1 Reproductive biology of *Pinus sylvestris*

*Pinus sylvestris* is a monoecious gymnosperm which bears male and female reproductive structures (strobili) separately on the same individual. Pollen production begins from the age of 10–15 years (Carlisle & Brown, 1968). The pollen grains, which have lateral air sacs to assist dispersal by wind, are borne on strobili which are highly variable in size, but are typically 30–60 mm in length. The pollen can retain high germinability rates after several days’ exposure to air (Lindgren & Lindgren, 1996). The dispersal kernel is strongly leptokurtic (Robledo‐Arnuncio & Gil, 2005), with the majority of pollen falling proximally, but infrequent long‐distance mating events do occur. Robledo‐Arnuncio (2011) reports that 4.4% of seedlings sampled from an isolated *P. sylvestris* stand in Iberia were sired by individuals in a stand which was c. 100 km away, suggesting that significant long‐distance dispersal of pollen was not rare in the sparsely forested landscape studied.

Female strobili are roughly 5–7 mm long and tend to be borne on the tips of well‐illuminated branches and can set seed in trees that are 6 years old or over (Carlisle & Brown, 1968). Female strobili are pollinated during summer. Pollen comes into contact with a liquid secretion from the female strobilus (“pollination drop”) and is drawn into the pollen chamber. The pollen chamber of *P. sylvestris* has room for around six pollen grains (Sarvas, 1962), and because grains are often clustered together so that more than one pollen grain may enter simultaneously, it has been suggested that early arriving pollen has a greater chance of occupying a position closest to the nucellus, increasing its probability of fertilizing the ovum (Sarvas, 1962). Varis, Santanen, Pakkanen, and Pulkkinen (2008) point out that the reality may be more complex than this, involving competitive interactions among pollen grains, for instance via genetic differences in the temperature requirements of pollen germination and the rate of pollen tube growth. Whilst self‐pollination can occur, little selfed seed is produced because it tends to abort due to presence of lethal homozygous recessives (Hedrick, Savolainen, & Kärkkäinen, 1999).

### 2.2 Selection of sites and individuals

**Figure 1:**
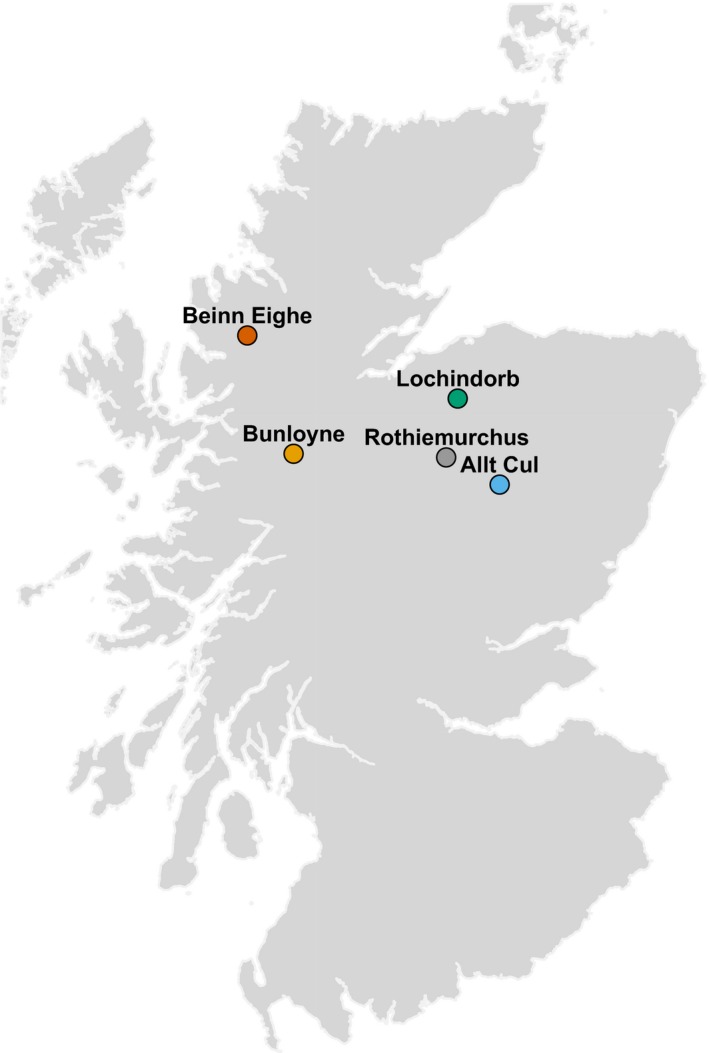
Map of mainland Scotland indicating the location of field sites

Selection of sites was based on an inventory of ancient, semi‐natural pinewoods in Scotland, which are considered to have persisted through natural regeneration since postglacial establishment and are known collectively as the “Caledonian pinewoods” (Forestry Commission, 1999). The site names applied here are those from the Caledonian pinewood inventory. In the first year of observation, three sites (Beinn Eighe, Rothiemurchus, and Allt Cul) were selected on the basis of their location along a longitudinal gradient (Figure 1), which in upland Scotland represents the most important axis of environmental variation, with highly oceanic (warm and wet) conditions in the west of the country and more continental (colder and drier) conditions in the east of the country (Barrow & Hulme, 1997). This gradient in continentality within Scotland has been shown to exhibit correlations with variation in phenotypic traits among *P. sylvestris* populations in common‐garden studies (Donnelly et al., 2016; Perry, Wachowiak, et al., 2016; Perry, Brown, et al., 2016; Salmela et al., 2011, 2013). These sites were deliberately chosen because they were geographically far apart yet were readily accessible by road such that all could be visited in a single round trip lasting 2 or 3 days. Hence, we incurred only a small offset in observation timing among sites, meaning that data could legitimately be compared (Figure 1). For 2015 and 2016, a further two sites (Lochindorb and Bunloyne) were added to the sample and were chosen because they also lay on a route which would not greatly extend the total period of observation and because they were thought to be intermediate in terms of long‐term average temperature compared to the three sites visited in 2014 (based on interpolated estimates of growing degree days for 1961–2000 (Perry & Hollis, 2005), Table 1). The maximum distance between these five populations is 137 km, a distance which can likely be occasionally achieved by wind dispersed pollen in certain conditions (Varis et al., 2009).

**Table 1:** Location details of each of the five field sites, indicating long‐term average growing degree days (GDD) as an indicator of temperature regimes and the years in which the sites were visited. Estimates for GDD are based on interpolation between weather stations which are projected onto 5 × 5 km grids for the whole of the UK (Perry & Hollis, 2005). Location and altitude are given for the geometric centroid of sampled trees

Site name	Latitude	Longitude	Altitude (m)	Average GDD	2014	2015	2016
Beinn Eighe	57.63	−5.36	90	1357.3	+	+	+
Rothiemurchus	57.15	−3.77	307	1046.3	+	+	+
Allt Cul	57.04	−3.35	475	558.3	+	+	+
Bunloyne	57.14	−4.95	150	687.6^a^		+	+
Lochindorb	57.4	−3.69	372	917.8		+	+

a The site at Bunloyne lies in a sheltered, low altitude area within a mountainous area, is immediately surrounded by hills and in an area with particularly low density of meteorological stations. Interpolated long‐term temperature values are considered not representative of the field site, which is expected to be much warmer than its surroundings.

Twenty trees within each site were selected along circuitous walking routes for inclusion within the sample. To minimize bias, a patch of trees would be identified from a distance and then the first one arrived at that was: accessible, seemingly of a reproductively mature age, amenable for visual inspection and likely to survive the three sampling years was marked nonpermanently for inclusion within the sample. No measurements of tree size or age were made of the sampled trees. Where possible, the recorded trees were separated by at least 100 m. However, at Bunloyne, Allt Cul, and Lochindorb, which are small sites containing fewer than 100 mature pine trees, some of the recorded trees were unavoidably less than 100 m apart. At these three small sites, most of the pine trees were very old and there were few young trees and almost no natural regeneration. Population sizes at the two larger sites of Rothiemurchus and Beinn Eighe were (orders of magnitude) larger, and age and size structure were more variable.

### 2.3 Phenological scoring

**Figure 2:**
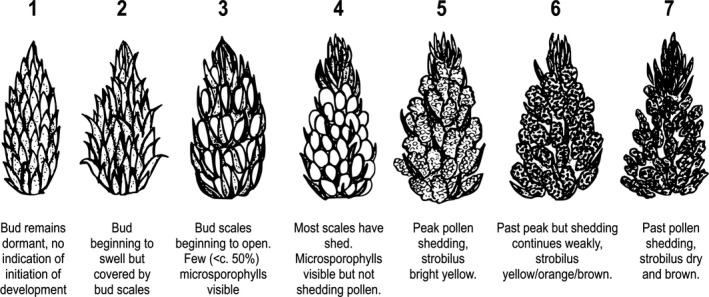
Line illustrations and descriptions indicating strobilus morphology at each of the seven modal states

At each site, the preselected sample of 20 trees was visited repetitively (approximately every 10 days) during the months of May and June in 2014, 2015, and 2016, in order to make phenological recordings during the period of male strobilus development. Strobili were assigned an ordinal developmental score, based on their morphology, which is an extension of a scale described by Gömöry, Bruchanik, and Paule (2000) (Figure 2).

Male strobili in pine trees are highly abundant, and so a pragmatic decision to score the five most developed strobili on each tree was made. These were scored based on a 1 min visual search of the entire crown either unaided or with binoculars. Tree branches were agitated to confirm whether pollen shedding was taking place. In almost all cases, the five most developed strobili were all at the same stage of development, although there can be considerable variation throughout the crown of a tree, particularly between north and south facing sides of the crown (Pérez, Martínez, Miranda, & Sánchez, 2002).

### 2.4 Climatic data

Daily maximum and minimum air temperatures for the nearest Met Office weather station to each recorded population were obtained from the first of January 2013 until the 30th of June 2016. Average daily temperature was calculated as the median of the maximum and minimum temperature. Daily average temperatures were then used to calculate indices of thermal time for the periods preceding anthesis. To do this, we calculated growing degree days (GDD), which is the cumulative daily sum of the number of degrees Celsius on days in which the average air temperature exceeds 5.5°C, beginning on the first of January in each year. This is a standard index of thermal time which has been found to be informative for understanding climatic cues of spring phenological activity in temperate trees (Murray, Cannell, & Smith, 1989; Vitasse & Basler, 2013), including *P. sylvestris* (Chung, 1981; Luomajoki, 1993).

It should be noted that there was wide variation in the distance between weather stations and sampling sites (Table S1), and in some cases, the temperatures observed at weather stations may not be particularly representative of those of the sampling site. This may be due not only to geographical distance but also to the effects of altitude and aspect, which vary at narrow spatial scales in the Scottish Highlands (Salmela et al., 2010). The nearest weather stations to Bunloyne and Lochindorb are particularly geographically distant and situated in different topographical contexts (Table S1). In the final year of observation, three small automated temperature recorders (iButton; Maxim Integrated Products, Sunnyvale, California, USA) were deployed at each of these two sites in order to confirm the disparity between local temperatures and weather station records. Variation in temperature within sites was not considered.

### 2.5 Statistical analyses

All the statistical analyses were performed in R version 3.2.3. (R Core Team, 2015). Data management, analysis, and visualization relied upon the “dplyr” (Wickham & Francois, 2015) and “ggplot2” (Wickham, 2009) packages.

Due to the time intervals between site visits, it was not always possible to be at each of the sites at precisely the time when the majority of pollen is shed, a period which, in Finland, lasts only around 3 days per tree (Parantainen & Pulkkinen, 2003). To overcome this, estimates of the differences in timing of development between sites were made using cumulative link models, a type of ordinal logistic regression implemented using the “ordinal” package within R (Christensen, 2015). The purpose of a cumulative link model is to estimate the cumulative probability that a given observation will fall into one of a series of ordinal categories based on predictor terms provided in model specification. A major advantage of ordinal regression models for this purpose is that they recognize that an ordinal response is bounded at both ends and make no assumption about the spacing between values of the response variable, as would be implied by a linear regression model with a continuous response (Harrell, 2015).

#### 2.5.1 Between site variation

In the cumulative link models, different intercepts for each factor level *j* (e.g., sites) were set as a function of a constant *Θ*, meaning that a common slope was applied to each *j*. This means that the slopes for different sites did not vary and as such, differences between sites were the same at any of the response levels (1–7).

Optimally, the phenological scores would be modeled thus

$$P_{\left[\mathrm{STROBILUS}==x\right]}=\mathrm{Day}+\mathrm{Site}\times \mathrm{Year}$$

In which *P*_[STROBILUS == x]_ is the phenological observation and x is any one of the phenological modal states (1–7). Day is the day of observation counting from May 1. Site and Year are factor variables.

As each of the sites was not visited every year, the full dataset is rank deficient. For this reason, the Site × Year interaction term was dropped and, to investigate interactions between site and year, separate models were fitted for each year and to a restricted dataset containing only the sites visited in a given year.

To estimate the time lag between sites, we followed the method of Vander Mijnsbrugge et al. (2015), using beta coefficients returned by the fitted models. The time lag is defined as the difference in number of days in which half of the strobili at one site has reached the same phenological stage as at another site and is calculated thus

$${\mathrm{Day}}_{[\mathrm{Site}\;i]}-{\mathrm{Day}}_{[\mathrm{Site}\;j]}=\left(\beta_{[\mathrm{Site}\;j]}-\beta_{[\mathrm{Site}\;i]}\right)/\beta \;\mathrm{Day}$$

in which β _[Site i, j]_ are the estimated beta coefficients for sites in the fitted model and β Day is the estimated coefficient for time. Confidence intervals for these estimates were calculated using nonparametric bootstrapping but were considered to be insufficiently stringent to account for the variation within sites and the time period over which the majority of pollen was likely to have been shed, which is noted to last for 3 days (Parantainen & Pulkkinen, 2003). To account for this variability, an additional 3 days were added to the confidence intervals for “significance” testing. If these penalized confidence intervals for any pairwise comparison among sites overlapped zero, the difference between sites was considered not significant.

#### 2.5.2 Between year variation

To investigate the differences in timing of phenological events among years, a similar model was fitted and was based on a restricted dataset including only the three sites which were visited in all three sampling years.

$$P_{\left[\mathrm{STROBILUS}==x\right]}={\text{Day}}_{\left[\mathrm{from}\;\mathrm{May}\;1\;(\mathrm{inclusive})\right]}+\text{Site}\times \text{Year}$$

#### 2.5.3 Thermal time response

To investigate male pollen phenological responses to indices of thermal time, models were fitted to indices of thermal time (growing degree days, GDD), rather than calendar dates. The temperature data for the nearest weather stations to Bunloyne and Lochindorb were considered likely to be unrepresentative of conditions at the two pinewood sites (Table S1), and these were therefore excluded from the analysis to concentrate on the extreme sites and an intermediate temperature site which had data from a weather station that was much nearer to it (Rothiemurchus). This model was specified thus

$$P_{\left[\mathrm{STROBILUS}==x\right]}=\text{GDD}+\text{Site}\times \text{Year}$$

#### 2.5.4 Variation within sites

To investigate the consistency across observation years in the rank order of trees’ male strobilus development at each site, the sum of strobilus scores for each tree was calculated on each visit and then ranked. The sum of rankings across each visit in each year was then calculated to give an overall impression of the order of development in each year, and these sums were then ranked for each site in each year, on the basis that the tree with the lowest summed rank strobilus score will develop earliest. Correlation among years was then tested on these ranked values with Spearman's rank correlation coefficient.

## 3 RESULTS

### 3.1 Variation in timing of strobilus development among sites

**Figure 3:**
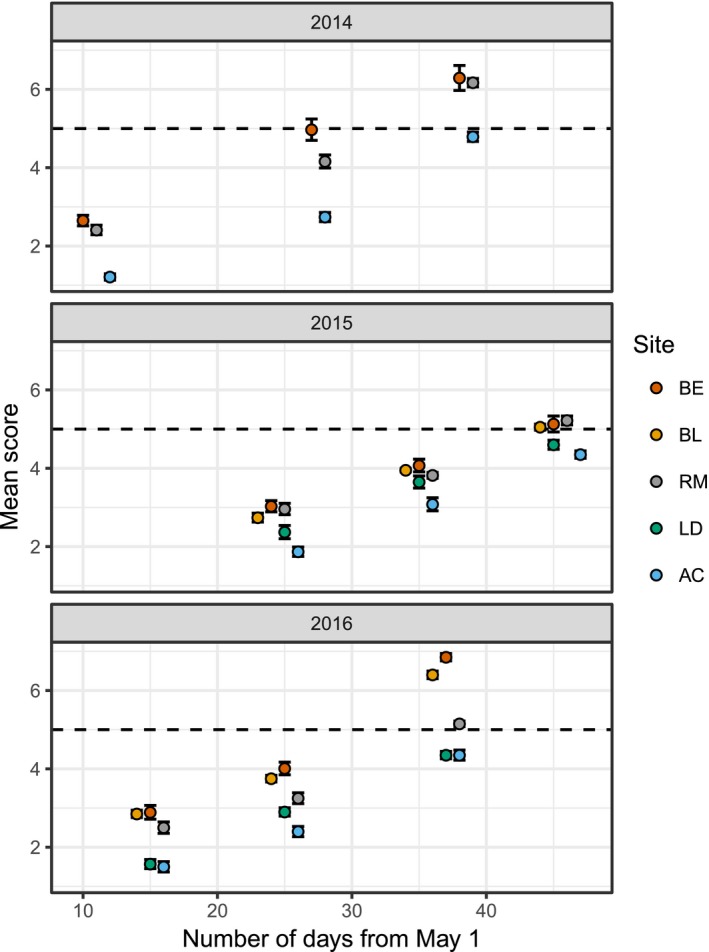
Mean strobilus scores and 95% confidence intervals on the observation dates. The dashed horizontal line is plotted at stage 5, which is when trees are at peak pollen production. Site abbreviations are AC, Allt Cul, BE, Beinn Eighe, BL, Bunloyne, LD, Lochindorb, RM, Rothiemurchus. Note that RM and BE overlap one another on the final date of observation in 2016

At the site level, there were clear differences in the timing of strobilus development between populations, with the most westerly site (Beinn Eighe) consistently developing earliest, and the most easterly site (Allt Cul), typically developing latest (Figure 3). The intermediate sites typically followed the same order with Bunloyne second, Rothiemurchus third, and Lochindorb fourth.

### 3.2 Predicting timing of pollen production

**Figure 4:**
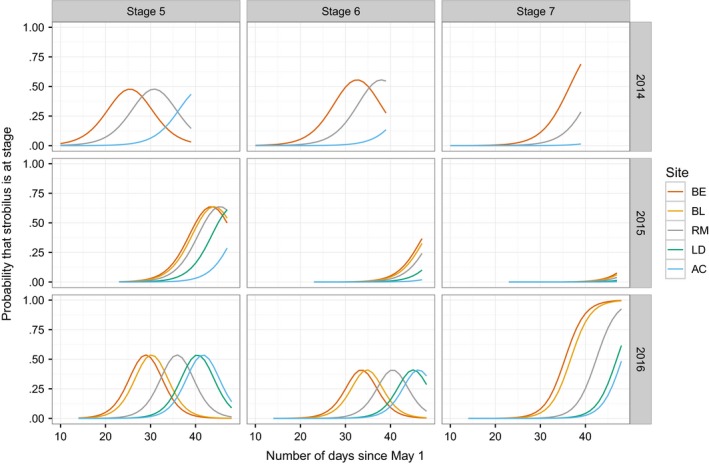
Modeled timing of pollen shedding indicating for each score level, exceeding those which come before pollen is shed (5–7), the probability that strobili of trees at each of the sites have reached a given score

**Figure 5:**
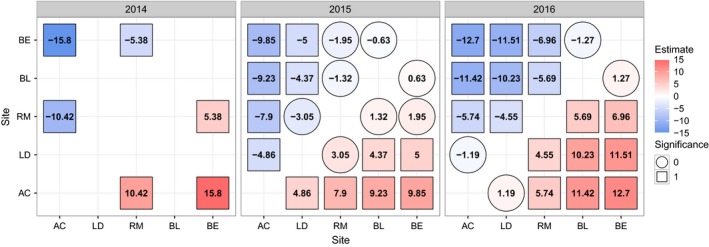
Comparison of estimated developmental time difference in days between sites in 2014, 2015, and 2016. Square symbols represent “significance,” which is defined as differences between sites which exceed 3 days plus the confidence interval of the site estimate

The cumulative link models found significant differences among sites (Figure 4, Table S2) and were used to generate parameter estimates to predict the time lag between sites (Figure 5). In each year, the greatest time lags were between Beinn Eighe (BE) and Allt Cul (AC), ranging from 9.85 days in 2015–15.8 days in 2014. Allt Cul and Lochindorb (LD) were separated from the other sites by more than 3 days in the years sampled, although the difference between Allt Cul and Lochindorb was less than 2 days in 2016 (Figure 5.).

Despite tendencies for these timing differences between sites, the model predicts overlap between the tails of the distributions for even the most distant sites (BE, AC) (Figure 4). For instance, in 2014, at the time when the latest 10%–15% of strobili were expected to be at stage 5 at Beinn Eighe, the earliest 10%–15% were predicted to be at stage five in Allt Cul (intersection of the red and blue curves on Figure 4). This means that, all else being equal, there is a possibility of pollen from Beinn Eighe arriving at Allt Cul at a time when some female strobili are receptive.

### 3.3 Variation in timing of strobilus development among years

**Figure 6:**
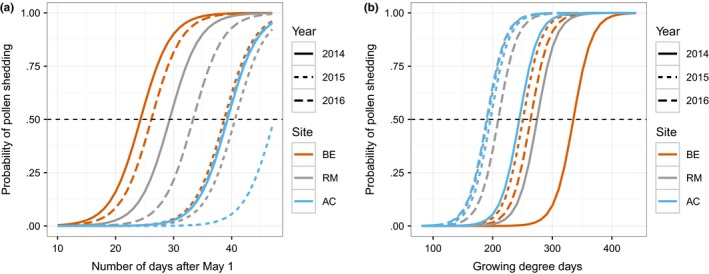
Modeled response of pollen shedding indicating the cumulative probability that strobili have minimally reached stage 5 (peak pollen shedding) based on the response to a) Time, represented by the number of calendar days from May 1 and b) accumulated growing degree days

Although the rank order of sites in terms of male strobilus development was consistent across years, the actual timing and the differences in timing between sites were variable between years in most cases. An exception is for Allt Cul, where the timing was the same in 2014 and 2016 (Figure 6a, Table S3).

### 3.4 Response of strobilus development to thermal time

When thermal time (GDD) was considered in place of calendar time, we found that the pattern was reversed whereby a lower heat sum has been accumulated at Allt Cul by the time trees are predicted to be shedding pollen than at Beinn Eighe (Figure 6b). However, as with calendar time, the degree day sum at the predicted time of pollen shedding varied by year (Table S4), suggesting that there was plasticity in the response and that development is not driven solely by spring temperature regimes.

**Figure 7:**
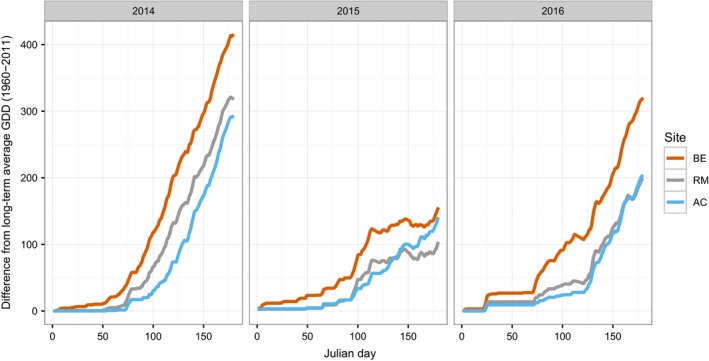
Differences from long‐term average GDD based on temperature data from the nearest weather stations 1960–2011

**Figure 8:**
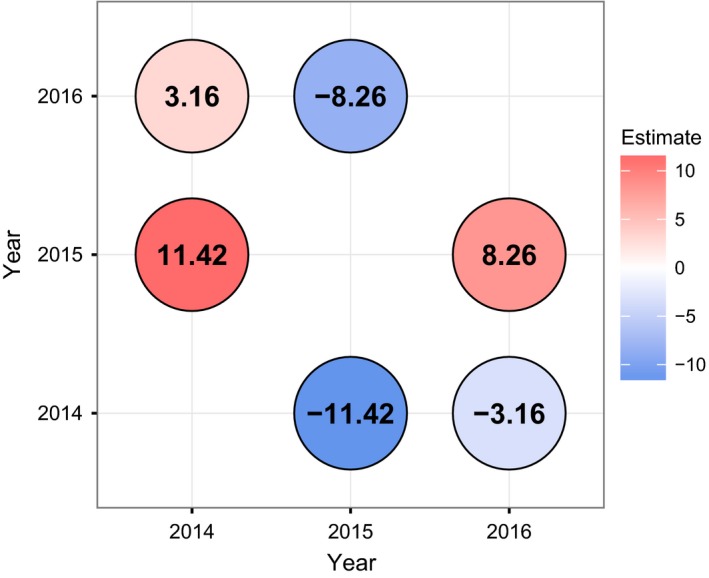
Estimated time lags/leads (number of days) between the different years, based on pooled estimates for AC, BE, and RM, as shown in Figure 6 and Table S3

Of the three sampling years, 2014 experienced the warmest temperatures in the period leading up to and including strobilus development (Figure 7). Correspondingly, development was earliest in this year, showing a tendency to take place 3.2 days earlier than in 2016 and 11.4 days earlier than 2015 (Figure 8). In each of the three sampling years, the greatest high temperature anomalies were observed at Beinn Eighe (Figure 7), suggesting that differences in asynchrony may be due to local anomalies rather than an effect of uniformly warmer conditions.

Interannual climatic variation also seems to influence the range of variation between populations. The range of variation between sites was greatest in the warmest year (15.8 days in 2014) and lowest in the coolest year (9.85 days in 2015) (Figure 8).

### 3.5 Variation within sites

Despite tendencies for earlier development in sites in the warmer west, there was considerable variation within sites. For instance, in 2014 and 2015, some of the trees at Beinn Eighe were reluctant to flower at all, containing very few or no male strobili. Trees were randomly chosen in early May 2014, before anthesis had begun. At that time, it was impossible to determine whether all of the trees were reproductively mature or active. It may be the case that the trees which did not reach advanced stages of development were sterile or immature at that time, despite deliberate attempts to choose trees which looked old enough to produce male strobili (c. 10–15 years in *Pinus sylvestris* (Carlisle & Brown, 1968)). Another example of a surprising result when within site variation is considered is that a single tree was shedding pollen at Lochindorb in 2015 before any of those at Bunloyne and Rothiemurchus, despite the general tendency for slower development at Lochindorb (Figure 3). This individual tree was again among the first at Lochindorb to shed pollen in 2016. The order of development of individual trees tended to be correlated in different years (Table 2, Fig. S1), with high Spearman rank coefficient values at Beinn Eighe (ρ = 0.69–0.88, *p *<* *.001) and Rothiemurchus (ρ = 0.72–0.78, *p < *.001). Correlation coefficient values were smaller or nonsignificant at Allt Cul (Table 2).

**Table 2:** Spearman rank correlation coefficients for the pooled sum of phenological rankings for each tree in each year. Strong correlations suggest that trees within a site develop in the same order in different years

Site	2014/2015	2014/2016	2015/2016
AC	0.47*	0.37n.s.	0.49*
BE	0.74***	0.69***	0.88***
BL			0.36n.s.
LD			0.66***
RM	0.72***	0.72***	0.78***

Significance codes, ^n.s.^ *p *>* *.05, *p <* *0.05, ***p <* *.001.

## 4 DISCUSSION

There were large differences in the predicted timing of peak pollen production between the sites sampled in each year and between years. The largest of these differences were observed between the pair of sites that were separated by the greatest geographical distance (Beinn Eighe and Allt Cul). Populations in the warmer west showed a strong tendency to shed pollen earlier than those in the colder east, but the populations in the east were capable of producing pollen at much lower temperature sums. These results show the opposite pattern from common‐garden experiments in which populations from the colder east tended to commence spring growth earliest (Salmela et al., 2013). The apparent negative correlation between common‐garden and in situ field observations follows a pattern of countergradient phenotypic variation (Conover & Schultz, 1995; Levins, 1969; Soularue & Kremer, 2012, 2014).

The size of these observed differences in the predicted timing of pollen shedding (9.85–15.8 days) suggests that direct pollen transfer between the extreme populations, which would already be infrequent due to the large distance between them, would be further limited by a degree of reproductive asynchrony. Nonetheless, the cumulative link models predicted a small overlap between the tails of the distributions of the reproductive period between the extreme populations and the ranking of individuals within sites tended to be correlated between years (particularly in BE and RM), which is a recognized phenomenon in *Pinus sylvestris* (Burczyk & Chalupka, 1997), and in several broadleaved tree species (Delpierre, Guillemot, Dufrêne, Cecchini, & Nicolas, 2016; Hinks et al., 2015). Due to rank correlation in the order of strobilus development within populations among years and a small degree of reproductive synchrony, pollen dispersal among distant populations would most likely lead to assortative mating between temporally overlapping subsets of each population. Assortative mating among the populations studied would involve immigrant alleles from the latest individuals to produce pollen in a warmer environment (BE), into a receiving environment which selects for early growth initiation (AC). The late warm‐adapted alleles may be maladaptive in the cold environment and therefore never recruited (Soularue & Kremer, 2012, 2014). The largest differences in timing of pollen production between sites were observed in 2014, which was the warmest sampling year. The smallest differences were observed in 2015, which was the coldest year. Notably, in each of these three years, temperatures in the western site (Beinn Eighe) were particularly high compared to long‐term averages, suggesting that spatially variable climatic warming (i.e., greater levels of warming in the west than elsewhere) may lead to increasing reproductive asynchrony among populations.

It is important to note that there are many other populations of *Pinus sylvestris* between those sampled here, which will presumably exhibit intermediate timing. Although synchrony was limited between the extreme sites (BE and AC), which are separated geographically by 137 km, the differences in timing of strobilus development between more proximal populations were smaller and, all else being equal, unlikely to impose an insurmountable barrier to reproduction between populations. Furthermore, the area of timber plantations of *Pinus sylvestris* in Scotland exceeds the area of semi‐natural woodlands by over five times (Mason, Hampson, & Edwards, 2004). The genetic base of such plantations is mixed, including material of unknown origin and material derived from seed orchards based on seed collected from phenotypically superior trees growing in Scotland and elsewhere (Lee, 2002). Effective gene flow between exotic‐origin plantations and native populations of *Pinus sylvestris* has been reported in southern Iberia (Ramírez‐Valiente & Robledo‐Arnuncio, 2015; Unger, Vendramin, & Robledo‐Arnuncio, 2014). The occurrence of gene flow between exotic and mixed origin plantations and semi‐natural populations in Scotland has not been tested but seems probable considering that *P. sylvestris* becomes reproductively mature before reaching rotation age (Ennos, Worrell, & Malcolm, 1998; Forrest & Fletcher, 1995; Salmela et al., 2010).

One shortcoming of the sampling regime is that only male strobili were observed. This was a practical decision which was made because male strobili are much more conspicuous than female strobili, being larger, abundant throughout the crown and with morphologies which are relatively easy to describe. In contrast, female strobili are much smaller and tend to be located higher up in the tree crown, in exposed, illuminated positions at the ends of branches (Carlisle & Brown, 1968) making their development difficult to record. *Pinus sylvestris* is thought to be protogynous, whereby female strobili are often receptive 1–3 days before male strobili shed pollen in seed orchards (Burczyk & Chalupka, 1997; Chung, 1981; Jonsson et al., 1976; Lindgren et al., 1995; Parantainen & Pulkkinen, 2003; Sarvas, 1962). However, there can be considerable temporal variation across a single tree crown. Pérez et al. (2002) report a delay of up to 1 week between the shaded and sunny sides of *Pinus pinaster* Aiton and *P. sylvestris* seems qualitatively similar. Nonetheless, the temporal difference between development of male and female strobili within a single tree crown is likely to exceed the differences within a branch and the variation within a population means that synchronous receptivity and pollen shedding within a large population will not be restricted due to protogyny.

However, if it is the case that some female strobili will be receptive before any local pollen is available, and there is an advantage to early pollination (Sarvas, 1962), it is more likely that nonlocal pollen contribution to any population will be from warmer than from colder environments. The prevailing winds in Scotland in May and June proceed from the southwest (Cook & Prior, 1987), meaning that there is a greater likelihood that pollen will be transported from the (warmer) west to the (colder) east. This directional bias in gene flow from warmer sites to colder sites may be beneficial in delivering alleles which would confer an adaptive advantage to seedlings produced under warmer temperatures predicted for the future (Aitken & Whitlock, 2013; Davis & Shaw, 2001), provided that the adaptive differences are not so great that selection for early development acts against these warm‐adapted alleles (Soularue & Kremer, 2012, 2014). Another consequence of this geographical variation is that the western populations are less likely to receive large volumes of nonlocal pollen than populations elsewhere. Collectively, native Scottish populations of *P. sylvestris* represent the westerly oceanic margin of the species’ natural range (Carlisle & Brown, 1968). Within Scotland, the western populations represent the upper temperature margin of Scottish populations, ostensibly the “rear edge” of the Scottish meta‐population in terms of gene flow, a pattern which is weakly supported by recent isolation‐by‐distance analyses of microsatellite data (González‐Díaz et al., *in prep*). The marginal status of these western populations and their potential capacity for contributing warm‐adapted alleles to other populations under climate change mean that they are important candidates for dynamic gene conservation (Fady et al., 2016; Hampe & Petit, 2005; Lefèvre et al., 2013).

## AUTHOR C ONTRIBUTIONS

RW, RE, JC, and SC conceived the ideas and designed methodology; RW and CRE collected the data; CRE produced Figure 2; RW analyzed the data with assistance from RE, JC, and SC. RW led the writing of the manuscript. All authors contributed critically to the drafts and gave final approval for publication.

## DATA ACCESSIBILITY

Raw data of pollen observations are available as supplementary information file data.csv.

## CONFLICT OF INTEREST

None declared.
