# Supplementary material for: Substantial variation in the timing of pollen production reduces reproductive synchrony between distant populations of Pinus sylvestris L. in Scotland
Source: Ecol Evol. 2017 Jun 15;7(15):5754–65. doi: 10.1002/ece3.3154 (PMC5586338; doi:10.1002/ece3.3154)

**Appendix 1.**

**Table S1**. Location details of each of the five field sites and their nearest weather stations for which daily temperature values were available. Distance, difference in altitude and the likely sign of difference in air temperature between site and weather station are also listed. Location and altitude are given for the geometric centroid of sampled trees

| Site name | Location (OSGB36) | Altitude (m) | Weather station name | Location  (OSGB36) | Weather station altitude (m) | Distance (km) | Altitude difference (m) | Temperature at site likely to be: |
| --- | --- | --- | --- | --- | --- | --- | --- | --- |
| Beinn Eighe | NG995654 | 90 | Kinlochewe | NH626629 | 19 | 4 | 71 | Slightly cooler |
| Rothiemurchus | NH930080 | 307 | Aviemore | NH896143 | 229 | 7.2 | 78 | Slightly cooler |
| Allt Cul | NO180953 | 475 | Braemar No. 2 | NO152919 | 341 | 4.5 | 134 | Cooler |
| Bunloyne | NH217097 | 150 | Cluanie Inn No. 3 | NH076117 | 218 | 14 | -68 | Warmer |
| Lochindorb | NH984355 | 372 | Cromdale | NJ072284 | 193 | 11.2 | 179 | Cooler |

**Table S2**. Estimated beta coefficients for parameters in the separate phenological models fitted for each year. Rothiemurchus, which is the intermediate site in most cases is the reference to which other sites are compared (estimated parameter for Rothiemurchus = 0).

| **Year = 2014** | | | | |
| --- | --- | --- | --- | --- |
|  | Estimate | Standard error | *z* value | Significance |
| Day | 0.320 | 0.013 | 25.29 | *** |
| Allt Cul | -3.334 | 0.191 | -17.44 | *** |
| Beinn Eighe | 1.721 | 0.165 | 10.43 | *** |
|  | | | | |
| **Year = 2015** | | | | |
|  | Estimate | Standard error | *z* value | Significance |
| Day | 0.365 | 0.012 | 29.72 | *** |
| Allt Cul | -2.887 | 0.181 | -15.968 | *** |
| Beinn Eighe | 0.712 | 0.174 | 4.088 | *** |
| Bunloyne | 0.483 | 0.168 | 2.882 | ** |
| Lochindorb | -1.113 | 0.172 | -6.461 | *** |
|  | | | | |
| **Year = 2016** | | | | |
|  | Estimate | Standard error | *z* value | Significance |
| Day | 0.451 | 0.012 | 36.93 | *** |
| Allt Cul | -2.587 | 0.163 | -15.87 | *** |
| Beinn Eighe | 3.140 | 0.184 | 17.04 | *** |
| Bunloyne | 2.566 | 0.166 | 15.46 | *** |
| Lochindorb | -2.051 | 0.156 | -13.11 | *** |

**Table S3**. Estimated beta coefficients and interaction terms in the phenological model fitted to investigate the timing of phenological development in different years. 2014 is the reference year to which other years are compared and Allt Cul is the reference site (estimated parameter for AC in 2014 = 0).

|  | Estimate | Standard error | *z* score | Significance |
| --- | --- | --- | --- | --- |
| Time | 0.351 | 0.007 | 47.624 | *** |
| SiteBE | 5.321 | 0.194 | 27.485 | *** |
| SiteRM | 3.522 | 0.172 | 20.492 | *** |
| Year2015 | -2.799 | 0.173 | -16.141 | *** |
| Year2016 | 0.073 | 0.156 | 0.465 | *n.s.* |
| SiteBE:Year2015 | -2.272 | 0.235 | -9.686 | *** |
| SiteRM:Year2015 | -1.121 | 0.223 | -5.036 | *** |
| SiteBE:Year2016 | -0.750 | 0.228 | -3.282 | ** |
| SiteRM:Year2016 | -1.434 | 0.214 | -6.698 | *** |

**Table S4**. Estimated beta coefficients and interaction terms in the phenological model fitted to investigate the effect of temperature accumulation (GDD) on phenological development. 2014 is the reference year to which other years are compared and Allt Cul is the reference site (estimated parameter for AC in 2014 = 0).

|  | Estimate | Standard error | *z* score | Significance |
| --- | --- | --- | --- | --- |
| GDD | 0.064 | 0.001 | 47.635 | *** |
| SiteBE | -5.807 | 0.223 | -26.056 | *** |
| SiteRM | -1.914 | 0.171 | -11.158 | *** |
| Year2015 | 3.095 | 0.172 | 17.982 | *** |
| Year2016 | 3.505 | 0.168 | 20.908 | *** |
| SiteBE:Year2015 | 2.320 | 0.242 | 9.577 | *** |
| SiteRM:Year2015 | 2.217 | 0.230 | 9.659 | *** |
| SiteBE:Year2016 | 1.088 | 0.236 | 4.616 | *** |
| SiteRM:Year2016 | 0.666 | 0.215 | 3.094 | *** |

**Fig. S1**. Rank order of tree development in each year, compared to a reference year of 2015 in which all sites were visited, based on ranked summed strobilus scores.


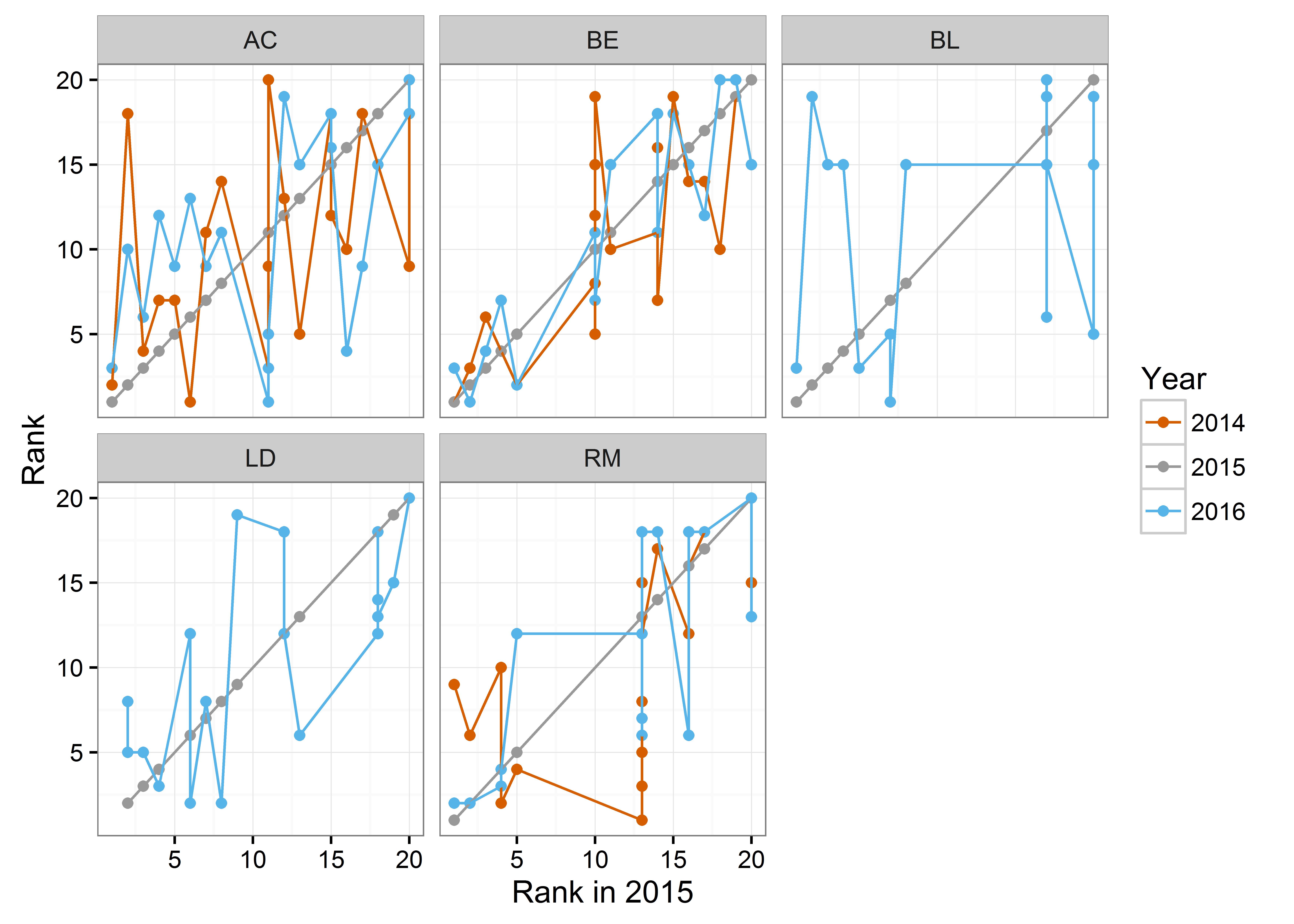

Supplement: Supplementary file 1 [file ECE3-7-5754-s001.docx]
